# Strain threshold for ventilator-induced lung injury

**DOI:** 10.1186/cc9618

**Published:** 2011-03-11

**Authors:** A Santini, A Protti, M Cressoni, T Langer, D Febres, G Conte, L Lombardi, M Lattuada, P Taccone, L Gattinoni

**Affiliations:** 1 Università degli Studi di Milano, Dipartimento di Anestesiologia e Terapia Intensiva, Milan, Italy; 2 Università degli Studi di Milano, Centro Ricerche Chirurgiche Precliniche, Milan, Italy; 3 Fondazione IRCCS Ca' Granda, Ospedale Maggiore Policlinico, Milan, Italy

## Introduction

Unphysiological lung strain (tidal volume/functional residual capacity, TV/FRC) may cause ventilator-induced lung injury (VILI) [1]. Whether VILI develops proportionally to the applied strain or only above a critical threshold remains unknown.

## Methods

In 20 healthy, mechanically ventilated pigs, FRC and lung weight were measured by computed tomography. Animals were then ventilated for up to 54 hours with a TV set to produce a predetermined strain. At the end, lung weight was measured with a balance. VILI was defined as final lung weight exceeding the initial one.

## Results

**Figure 1:**
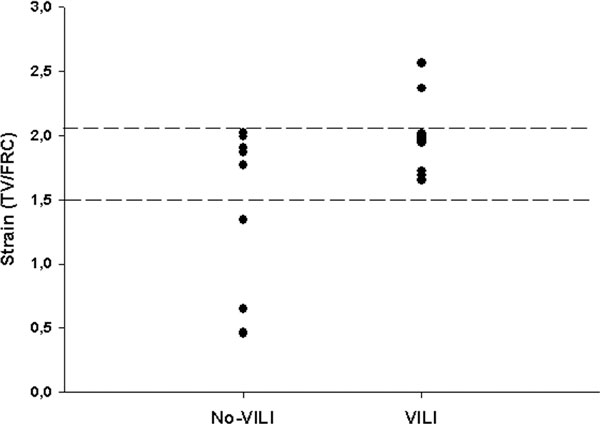


Lung weight either did not increase at all (no-VILI group; lung weight change -73 ± 42 g, *n *= 9) or markedly augmented (VILI group; 264 ± 80 g, *n *= 11). In the two groups, strain was 1.38 ± 0.68 and 2.16 ± 0.50 (*P *< 0.01), respectively. VILI occurred only when lung strain reached or exceeded a critical threshold, between 1.5 and 2.1 (Figure 1).

## Conclusions

In animals with healthy lungs VILI only occurs when lung strain exceeds a critical threshold.
